# Cellular infiltrate in cutaneous leishmaniasis lesions and therapeutic outcome^☆^^☆☆^

**DOI:** 10.1016/j.abd.2021.02.006

**Published:** 2021-07-28

**Authors:** Camila Sampaio Ribeiro, Riam Rocha França, Juliana Almeida Silva, Silvana Conceição da Silva, Sílvia R.B. Uliana, Viviane Sampaio Boaventura, Paulo R.L. Machado

**Affiliations:** a Universidade Federal da Bahia, Salvador, BA, Brazil; b Immunology Service. Hospital Universitário Prof. Edgar Santos. Universidade Federal da Bahia, Salvador, BA, Brazil; c Leishmaniasis Laboratory, Department of Parasitology, Instituto de Ciências Biomédicas, Universidade de São Paulo, São Paulo, SP, Brazil

**Keywords:** CD4 antigens, CD8 antigens, Immunohistochemistry, Interleukin-17, Leishmaniasis, cutaneous, Plasma cells

## Abstract

**Background:**

The treatment of cutaneous leishmaniasis is a challenge. A better understanding of the in situ mechanisms involved in the evolution and cure of the disease is essential for the development of new therapies.

**Objective:**

Correlate histopathological and immunological characteristics of cutaneous leishmaniasis lesions with clinical outcome after different treatment regimens.

**Methods:**

The authors analyzed cellular infiltration and immunohistochemistry staining for CD4, CD8 and IL-17 in biopsy samples from 33 patients with cutaneous leishmaniasis before treatment. All patients were recruited in a randomized clinical trial at Corte de Pedra (Bahia-Brazil) and assigned to receive Glucantime®, Glucantime® + Oral Tamoxifen or Glucantime® + Topical Tamoxifen. Patients were followed for 2 to 6 months to define disease outcome.

**Results:**

A similar expression of CD4, CD8 and IL-17 was observed in lesion samples regardless of clinical outcome. In general, a higher amount of CD8 cells were observed compared with CD4 cells. An important observation was that all patients whose cellular infiltrate did not contain plasma cells were cured after treatment.

**Study limitations:**

Isolated quantification of TCD8 and IL-17 using immunohistochemistry is insufficient to analyze the role of these molecules in the immunopathogenesis of cutaneous leishmaniasis. In addition, the expansion of the immunohistochemistry panel would allow a more complete analysis of the immune response in situ.

**Conclusions:**

The absence of plasma cells in cutaneous leishmaniasis lesions was related to a favorable therapeutic outcome.

## Introduction

Cutaneous Leishmaniasis (CL) is caused by an intracellular protozoan of the genus Leishmania transmitted to humans by inoculation through a mosquito bite. CL is endemic in South America and Brazil is amongst the ten countries that concentrate 75% of the cases of CL worldwide.^1^ It is typically a low-income country disease associated with poverty and unsanitary conditions.^2^

CL has a complex immunopathogenesis that has not been fully understood. It is well established that, in general, the main defense mechanism against intracellular agents, such as Leishmania, is associated with activation of cellular immunity through the production of Th1 cytokines such as IFN-γ, TNF-α and IL-12.^3^ In contrast, humoral immunity-related to Th2 cell response is associated with the persistence of intracellular organisms and thus, the progression of the infection.^4^ Therefore, Th1 immunity response would be adequate and effective to destroy parasites and avoid disease or promote a clinical cure. However, several data indicate that an exaggerated and unbalanced Th1 response may lead to intense tissue damage and more severe clinical forms of the disease.^5^, ^6^ In fact, it has been demonstrated that the ulcer size was positively correlated with circulating Th1 cytokines (IFN-γ and TNF-α) and activated T cells, which corroborates with this assumption.^7^

Additionally, it has been demonstrated an in-situ predominance of Th1 inflammatory cytokines (IFN-γ and IL-12) than anti-inflammatory ones (IL-10 and IL-4) before treatment of CL as well as a significant reduction of circulating TNF-α and IFN-γ levels after therapy and healing.^6^, ^8^ It has also been shown an association between cells expressing IL-17 and the intensity of the inflammatory infiltration.^9^

It is evident the important role of the immune system in the pathogenesis of CL and its implications for a definitive cure. Consequently, understanding the evolving local inflammatory response and its correlation to the healing of CL through characterization of the immune pattern at the lesion site may function as a therapeutic outcome predictor. The objective of this study was to verify if the cellular composition of the inflammatory infiltrates as well as CD4, CD8 and IL-17 expression at lesion site may correlate with therapeutic outcome in CL, a data not yet explored in the literature.

## Methods

### Population studied and follow-up

Patients were referred to the health post of Corte de Pedra, Bahia, Brazil, an endemic area of *Leishmania braziliensis* transmission. Patients included had an age of 18–65 years; diagnosis of CL between 30 and 90 days from the beginning of the cutaneous lesion; 01–05 ulcers with diameter, on its longest axis, between 10–50 mm. The diagnosis was confirmed through either histopathological examination showing amastigotes or by positive PCR for *L. braziliensis*. The exclusion criteria were pregnancy or breastfeeding, other associated acute or chronic illness, and previous leishmaniasis. Patients who met the entry criteria were randomly allocated to 3 groups: parental Glucantime® (Sb^V^) plus oral tamoxifen (OT), Sb^V^ plus topic tamoxifen (TT) or Sb^V^ only (control group). Patients were followed up monthly during 6 months to define therapeutic outcome; the details of the trial are described elsewhere.^10^

### Ethical aspects

The study was approved by the Ethics Committee of the Hospital Universitário Professor Edgard Santos in Bahia-Brazil (Research protocol nº 106/2009) and by the Ethics Committee of the University of São Paulo (Research protocol nº 993.562). Written informed consent was obtained from all patients included in the study.

### Drug administration

Meglumine antimoniate (Glucantime®, Aventis) was administered intravenously at a dose of 20 mg Sb^V^/kg/day for 20 days; oral tamoxifen (Sandoz) at a dosage of 20 mg twice a day during 20 days; tamoxifen 0.1% cream (compound drug) applied over all ulcers twice a day during 20 days (without dressing).

### Efficacy assessment

The initial and definite cure was defined as complete cicatrization of the lesion(s) without any infiltration in the borders, 2 and 6 months after the end of treatment, respectively.

### Biopsy and histopathology

A 4-mm punch biopsy was performed from the borders of the ulcers under local anesthesia prior to therapy. Fragments were fixed in a 10% formalin solution. Skin biopsies samples underwent routine histological processing and slides were stained with Hematoxylin and Eosin (H&E) for histopathological evaluation (inflammation pattern – perivascular or nodular and diffuse – the presence of necrosis, and cellular infiltrate composition: neutrophils, plasma cells, lymphocytes, eosinophils, histiocytes, epithelioid cells, giant cells, as well as the presence of amastigotes).

### Immunohistochemistry

An immunohistochemical study was performed as previously described on 33 biopsies.^11^ The following primary antibodies were used: rabbit anti-IL-17 (Santa Cruz Biotechnology, Santa Cruz, CA, USA) and mouse anti-CD8 (Dako, Carpinteria, California, USA), mouse anti-CD4 (Dako, Carpinteria, California, USA). Biotin-labeled anti-rabbit, anti-mouse IgG (DAKO HRP Advance – K 4068) was used as a secondary antibody. Digital images from all immunohistochemical-stained sections were acquired using an optical microscope (Nikon E-600) coupled to a digital Q-color 1 camera Olympus at 400× magnification. The expression levels of TCD4, TCD8 and IL-17 at the infiltrate were analyzed in five continuous fields per fragment and quantified by measurement of the area of stained cells/total area in the field. The first field selected for quantification on each specimen was the one containing visually more immunostained cells. Quantification of stained areas was performed blindly by two independent readers (CSR and VB) using Image-Pro Plus software (Media Cybernetics) and the result was expressed as median (quartile 25 to quartile 75). The results were correlated with these variables: area of the lesion, area of the lymphadenopathy, duration of the disease at day 0, and therapeutic outcome at 6 months.

Statistical analyses. Statistical analyses were performed using SPSS® version 20 for MAC® and also Prism 4 (GraphPad Software, San Diego, CA, USA). Differences were considered significant when p < 0.05. Continuous variables were expressed as median and the difference between the three groups was calculated by the Kruskal-Wallis Test and between two groups by the Mann-Whitney Test. Categorical variables were expressed as absolute frequency and relative frequency and to calculate the difference between three groups the Chi-Square Test was used and between two groups the Exact Fisher Test was performed. Cure rates were calculate using both Intention-To-Treat (ITT) and Per Protocol (PP) analysis.

## Results

### Therapeutic outcomes

The 38 CL patients were allocated into three groups: Sb^V^ (n = 15); Sb^V^+TT (n = 11) and Sb^V^+OT (n = 12) (Table 1). During follow-up, 04 patients did not complete correctly the treatment protocol due to: adverse reaction (02 patients: one from the Sb^V^+TT group and the other from the Sb^V^+OT group), use of Sb^V^ subdose (01 patient from Sb^V^+OT group), and 01 patient from the control group did not return to medical evaluations after inclusion. These patients were excluded from this in situ study. In addition, one patient of the clinical trial was not submitted to skin biopsy and was also excluded from this analysis. Thereby, among 38 patients of the clinical trial, 33 were part of this in situ study. Although not significant, the highest cure rates of 80% and 70%, were observed in the group Sb^V^+OT, at 2 and 6 months respectively (Table 1).

**Table 1:** Therapeutic outcome 2 months and 6 months after the end of the treatment by treatment group.

	Sb^V^	Sb^V^ + TT	Sb^V^ + TO	p-value
	(n = 14)	(n = 10)	(n = 10)	
**Cure rate at 2 months**	8 (57%)	5 (50%)	8 (80%)	ns^a^
**Cure rate at 6 months**	6 (43%)	4 (40%)	7 (70%)	ns^a^

a Pearsons Chi-Square test (comparison between all three groups and also between groups in pair); ns, not statistically significant.

### Immunological markers

**Figure 1:**
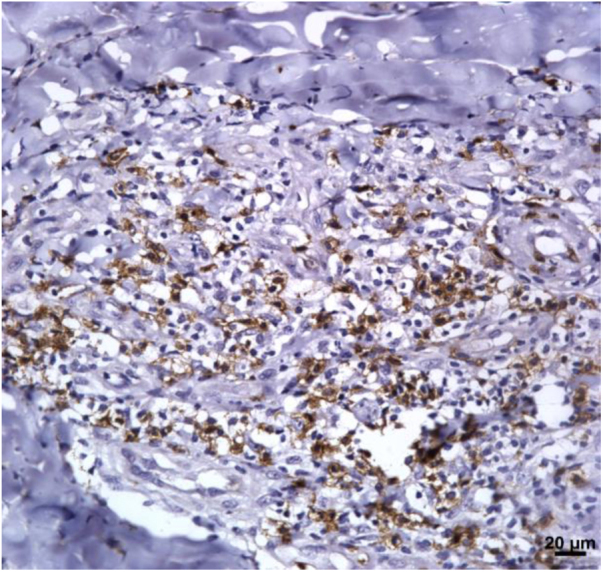
Skin fragment of CL ulcer showing immunohistochemical reaction evidencing CD8^+^ T-cells (×400).

**Figure 2:**
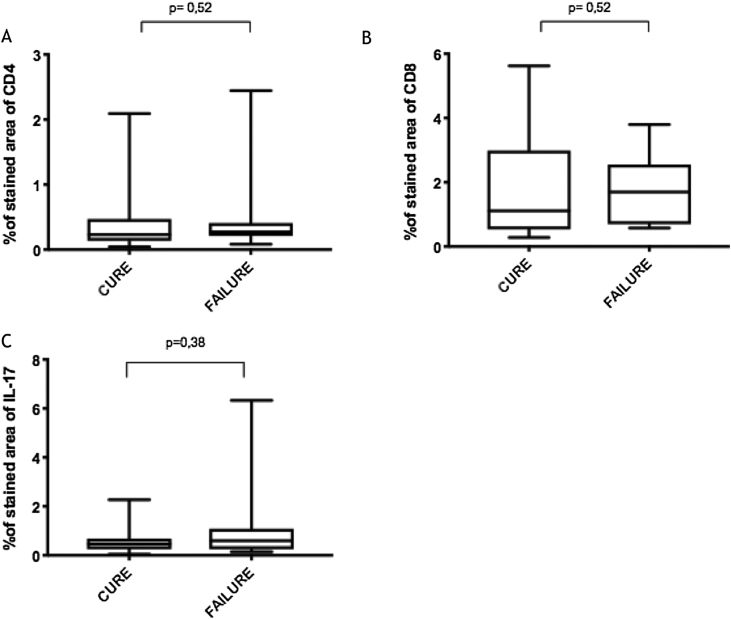
(A), CD4^+^ T; (B), CD8^+^ T; (C), IL-17 expression in healed and persistent lesions 2 months after treatment.

**Figure 3:**
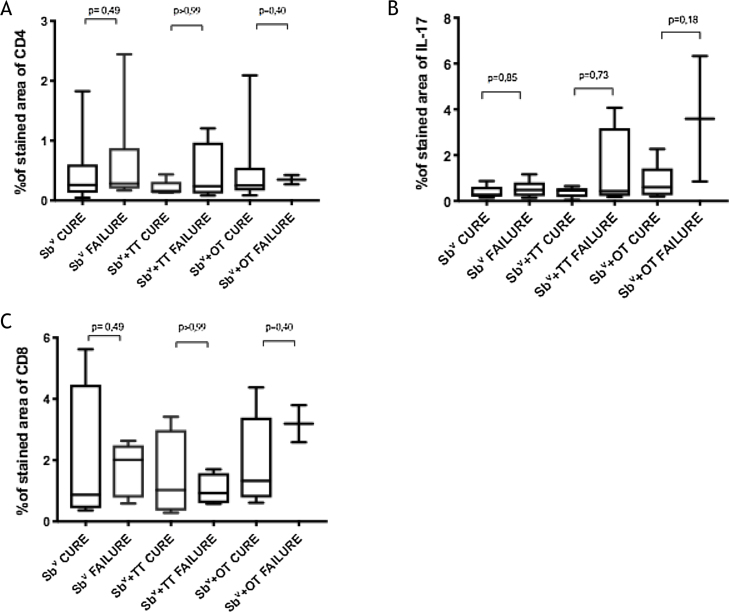
Percentage of the marked area of (A), CD4^+^ T; (B), IL-17; (C) CD8^+^ T in patients who healed and those who failed 2 months after the end of the treatment separated by treatment group.

The expression of CD8^+^ T in CL lesions before treatment is showed in Fig. 1. The analysis of the mean percentage of positively immunostained CD4^+^ T and CD8^+^ T-cells (Fig. 2) showed a higher expression of CD8^+^ T-cells. For both subpopulations, there were no differences in their expression between patients who healed (n = 17) and those who presented failure (n = 16) at 2 months. IL-17 immunostaining was higher in the failure group, however, there was no significant difference between cure and failure groups (Fig. 2). In addition, the evaluation of the expression of IL-17, CD4^+^T and CD8^+^ T-cells showed no differences when the authors compared the treatment subgroups (Fig. 3).

### Histopathological aspects

**Figure 4:**
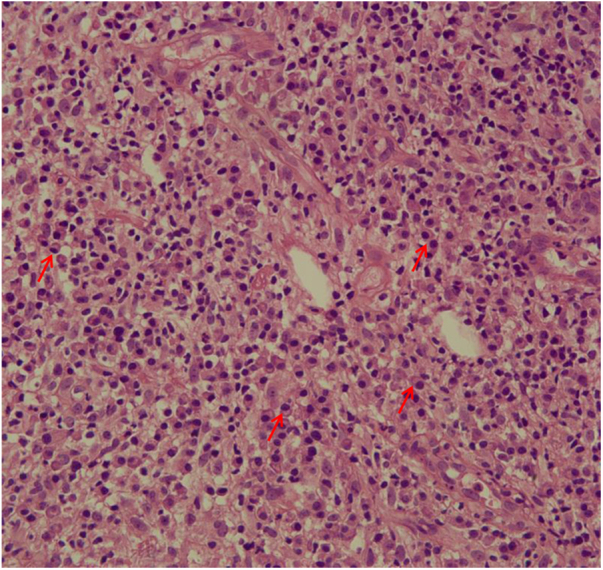
Plasma cells (red arrows) in the dermis of a fragment of skin biopsy of a CL ulcer (×400).

**Figure 5:**
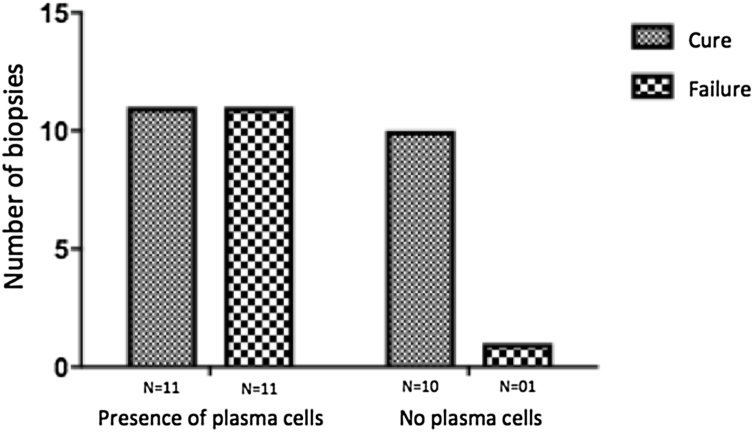
Plasma cells and therapeutic outcome 6 months after the end of the treatment in CL patients. * p < 0.05 (Fisher’s exact test).

The evaluation of the inflammatory infiltrates showed few differences among the cases. Most patients had biopsies with nodular and diffuse inflammation patterns and only 02 patients presented a superficial and deep perivascular pattern. In all cases, the predominant inflammatory cells were macrophages and lymphocytes. The majority had neutrophils 26/33 (78%) and in 14/33 cases (42%), areas of necrosis were observed in the dermis. Giant cells were visualized in 13/33 (39%) of lesions. In 3 out of 33 fragments analyzed, a significant amount of eosinophils was observed. Plasma cells were found in inflammatory infiltrate of the majority of CL patients (Fig. 4). Interestingly, plasma cells were absent in 11 out of 33 subjects (33%), and all those 11 patients were cured at 2 months, with only one relapse before 6 months (p < 0.05) (Fig. 5).

## Discussion

Pathogenesis of CL follows a complex set of interactions between many factors triggered by the host’s innate and acquired immune responses. These inflammatory responses mediate infection outcome and disease expression.^12^

The role of CD8^+^ T-cells in Leishmania infection may be associated with protection or tissue damage depending upon the effector functions triggered by the infection.^13^ However, there has been increasing evidence of the association of CD8^+^ T-cells with disease progression and pathology. There is a higher frequency of CD8^+^ T-cells in CL ulcers compared to early CL lesions before ulceration; CD8^+^ T-cells from ulcerated CL patients produced more granzyme B and had more cytotoxic activity than the cells from patients with subclinical infection; CD8^+^ T-cell-mediated cytotoxicity activates the NLRP3 inflammasome in CL lesions.^14^, ^15^, ^16^

In the present study, the authors observed a predominance of CD8^+^ T-cells over CD4^+^ T-cells in the pre-treatment ulcerated lesion. This observation strengthens the idea that CD8^+^ T-cells are more involved in the pathogenesis of the disease than CD4^+^ T-cells. However, we were not able to associate the expression of CD8^+^ or CD4^+^ T-cells to therapeutic outcomes.

The authors of the present study have also evaluated IL-17 expression due to its direct association with the inflammatory infiltrate on CL and its possible role in pathology.^9^ Although its highest expression was found in a CL subject that failed therapy, the comparison between the groups showed no differences. It is possible that a higher sample size as well as the evaluation of other inflammatory mediators like Il- or granzyme, could contribute to evaluating the CD8^+^ T profile present in the lesions and more accurately provide correlation with therapeutic outcome.

A striking data was the correlation between the absence of plasma cells in the ulcer before treatment and therapeutic cure at 2 months in all subjects, although one patient relapsed between 2 and 6 months. The presence of plasma cells is recognized as an important feature of the inflammatory infiltrate in CL. However, the exact role of plasm cells in CL pathogenesis remains to be determined and it has probably been underestimated. Ridley et al. noted a significant positive correlation between the presence of necrosis and intensity of plasma cell and lymphocyte infiltration.^17^ On the other hand, skin biopsies of non-ulcerated or atypical CL lesions, presumably with a lower inflammatory reaction, presented also a smaller amount of plasma cells.^18^ In the late stages of the CL lesion, a large number of plasma cells are typically present.^19^ Limitations of the study are the low number of patients and the absence of the evaluation of other markers of immune response in situ.

## Conclusion

The present study’s data suggest a possible role for plasma cells in the pathology of CL and as a marker of therapeutic outcome, since their absence in the infiltrate was associated with cure in all cases with only one relapse. Further evaluation of specific markers for B cells and plasmocytes in CL ulcers as well as functional studies should be performed to verify its potential pathogenic role in CL.

## Financial support

Funaderm– Fundo de Apoio à Dermatologia.

## Authors' contributions

Camila Sampaio Ribeiro: Approval of the final version of the manuscript; critical literature review; data collection, analysis, and interpretation; intellectual participation in propaedeutic and/or therapeutic management of studied cases; manuscript critical review; preparation and writing of the manuscript.

Riam Rocha França: Approval of the final version of the manuscript; data collection, analysis, and interpretation; preparation and writing of the manuscript; statistical analysis.

Juliana Almeida Silva: Approval of the final version of the manuscript; data collection, analysis, and interpretation; preparation and writing of the manuscript.

Silvana Conceição da Silva: Approval of the final version of the manuscript; data collection, analysis, and interpretation; preparation and writing of the manuscript.

Sílvia R.B. Uliana: Intellectual participation in propaedeutic and/or therapeutic management of studied cases; data collection, analysis, and interpretation.

Viviane Sampaio Boaventura: Approval of the final version of the manuscript; critical literature review; effective participation in research orientation; intellectual participation in propaedeutic and/or therapeutic management of studied cases; manuscript critical review; preparation and writing of the manuscript.

Paulo R. L. Machado: Approval of the final version of the manuscript; critical literature review; data collection, analysis, and interpretation; effective participation in research orientation; intellectual participation in propaedeutic and/or therapeutic management of studied cases; manuscript critical review; preparation and writing of the manuscript; study conception and planning.

## Conflicts of interest

None declared.
